# The association of neutrophil-lymphocyte ratio and lymphocyte-monocyte ratio with 3-month clinical outcome after mechanical thrombectomy following stroke

**DOI:** 10.1186/s12974-020-01739-y

**Published:** 2020-02-18

**Authors:** Danielle Lux, Vafa Alakbarzade, Luke Bridge, Camilla N. Clark, Brian Clarke, Liqun Zhang, Usman Khan, Anthony C. Pereira

**Affiliations:** 1 Department of Neurology, St George’s University Hospitals NHS Foundation Trust, London, SW17 0QT UK; 2 Department of Neurology, Royal Cornwall Hospitals NHS Trust, Truro, TR1 3LQ UK

**Keywords:** Neutrophil-lymphocyte ratio, Lymphocyte-monocyte ratio, Stroke, Mechanical thrombectomy

## Abstract

**Background and aim:**

Neutrophil-lymphocyte ratio (NLR) and lymphocyte-monocyte ratio (LMR) are associated with clinical outcomes in malignancy, cardiovascular disease and stroke. Here we investigate their association with outcome after acute ischaemic stroke treated by mechanical thrombectomy (MT).

**Methods:**

Patients were selected using audit data for MT for acute anterior circulation ischaemic stroke at a UK centre from May 2016–July 2017. Clinical and laboratory data including neutrophil, lymphocyte and monocyte count tested before and 24 h after MT were collected. Poor functional outcome was defined as modified Rankin Scale (mRS) of 3–6 at 3 months. Multivariable logistic regression analyses were performed to explore the relationship of NLR and LMR with functional outcome.

**Results:**

One hundred twenty-one patients (mean age 66.4 ± 16.7, 52% female) were included. Higher NLR (adjusted OR 0.022, 95% CI, 0.009–0.34, *p* = 0.001) and lower LMR (adjusted OR − 0.093, 95% CI (− 0.175)−(− 0.012), *p* = 0.025) at 24-h post-MT were significantly associated with poorer functional outcome when controlling for age, baseline NIHSS score, infarct size, presence of good collateral supply, recanalisation and symptomatic intracranial haemorrhage on multivariate logistic regression. Admission NLR or LMR were not significant predictors of mRS at 3 months. The optimal cut-off values of NLR and LMR at 24-h post-MT that best discriminated poor outcome were 5.5 (80% sensitivity and 60% specificity) and 2.0 (80% sensitivity and 50% specificity), respectively on receiver operating characteristic curve analysis.

**Conclusion:**

NLR and LMR tested at 24 h after ictus or intervention may predict 3-month functional outcome.

## Introduction

Inflammation has been increasingly recognised as a key contributor to the pathophysiology of acute ischaemic stroke (AIS) [1]. Elements of the immune system are intimately involved in the initiation and propagation of ischaemic brain injury, and developing immunosuppression secondary to cerebral ischaemia may possibly promote intercurrent infections [1]. Neutrophil to lymphocyte ratio (NLR) and lymphocyte to monocyte ratio (LMR) are potential novel biomarkers of baseline inflammatory response which have recently been reported as important predictors of AIS morbidity and mortality [2–4].

Randomised controlled trials have demonstrated that AIS treatment by mechanical endovascular therapy (MT) in addition to intravenous (IV) recombinant tissue plasminogen activator (rtPA) significantly improves the outcomes of AIS with large vessel occlusion [5]. Less favourable responses to MT were associated with advanced age, high baseline National Institutes of Health Stroke Scale (NIHSS) score, large infarct volume, recanalisation and poor cerebral collateral circulation [6–8]. Similarly, there is growing evidence that higher admission NLR may contribute to worse outcome at 3-month post-AIS treated with IV rtPA and/or MT [4, 9]. Conversely, lower lymphocyte to monocyte ratio (LMR) was associated with a poor prognosis in AIS including those treated with thrombolysis [10].

As part of auditing our thrombectomy outcomes, we noted whether there was a correlation between NLR and LMR and outcome in our cohort of AIS patients who underwent thrombectomy. We also investigated whether there were dynamic changes in NLR and LMR values and trends between NLR-stroke and LMR-stroke correlations.

## Methods

We performed a retrospective audit of consecutive, prospectively collected, ischaemic stroke cases referred for MT within a single regional, hyperacute stroke unit at St George’s Hospital. This is the main referral centre for MT in the UK, operating 24 h a day, 7 days a week. AIS patients admitted from 1st May 2016–1st July 2017 were used in the analysis. The patients’ data were entered in an audit database. Patients were selected for this investigation if they met all of the following criteria: adults (i.e. older than 16 years) (1) with a clinically confirmed acute anterior circulation ischaemic stroke with large vessel occlusion (2) undergoing MT. Exclusion criteria constituted (1) clinically confirmed acute posterior circulation ischaemic stroke; (2) patients with a history of terminal cancer, haematological disease, recent major trauma or surgery, severe hepatic or renal disease determined by clinical history or laboratory data; (3) immunosuppressant use; (4) active infections within the 2 weeks prior to admission.

Clinical data collected included demographics, vascular risk factors and admission baseline NIHSS score (determined by an in-house neurologist). Treatment parameters included IV rtPA administration and the Modified Thrombolysis in Cerebral Infarction (mTICI) grade (determined by an in-house interventional neuroradiologist: complete recanalisation classified as mTICI score 2b or 3 [11]), computed tomography (CT) angiography based cerebral collateral circulation [12] (good defined as more than 50% of the middle cerebral artery (MCA) territory vs poor) and stroke volume (< 1/3 MCA territory, > 1/3 MCA territory and larger than the MCA territory), anaesthetic mode (general anaesthesia vs local or conscious sedation) and haemorrhagic conversion based on the European Cooperative Acute Stroke Study (ECASS) classification [13]. The Alteplase Thrombolysis for Acute Noninterventional Therapy in Ischemic Stroke (ATLANTIS)/CT Summit criteria [14–16] has defined ‘> 1/3 MCA territory’ stroke volume as substantial involvement of ≥ 2 of the following 4 areas: frontal, parietal, temporal, or both basal ganglia and insula. Involvement of all 4 areas: frontal, parietal, temporal, basal ganglia, insula and beyond was defined as ‘beyond MCA territory’. All remaining scans were categorised as < 1/3 MCA involvement. All neuroimaging ratings were done by a neurologist (DL, UK and ACP). Outcome was measured by the modified Rankin Scale (mRS) at 90 days during clinical follow-up by trained staff. Poor outcome was defined as functional dependence and mortality (mRS 3–6), whereas good outcome was defined as an mRS score 2 or lower. Venous blood sampling was obtained on admission and within 24 h post-MT. Laboratory data included full blood count with white blood cells differentials, urea and electrolytes, liver function tests and C-reactive protein.

Statistical analysis was performed in SPSS (V.22; SPSS Inc., Chicago, IL, USA). Depending on the normality of distribution as assessed by the Kolmogorov-Smirnov test, continuous variables were compared using the *t* test for independent samples, or the Mann-Whitney *U* test. Categorical variables were analysed as frequency and percentage and differences among these variables were assessed by the chi-square test. For univariate correlation analysis, Spearman Rho was used. Logistic regression analysis was used to analyse the ability of NLR or LMR to predict 90-day mRS alongside other variables. The level of significance for these descriptive comparisons was established at 0.05 for two-sided hypothesis testing. Receiver operating characteristic (ROC) curves were used to test the overall discriminative ability of the NLR or LMR for outcome and to establish optimal cut-off points at which the sum of the specificity and sensitivity was highest.

## Results

A total of 121 patients met the criteria for inclusion and subsequent analysis. The mean age of the patient cohort was 66.4 years (SD ± 16.7) with 52% being female. Median baseline NIHSS score was 19 (range 1–28). Median baseline and 90-day mRS were 0 (IQR 4) and 3 (IQR 2), respectively. Ninety-four patients (77.6%) received intravenous rtPA as well. Complete recanalisation was achieved in 90 (74%) patients. Of the 25 (21%) patients with intracranial haemorrhage (ICH), 11 (9%) had symptomatic (sICH). Median NLR at admission (a_NLR) was 2.4 (range 0.5–31.8); LMR at admission (a_LMR) was 3.1 (range 0.6–8.6); 24-h NLR (24h_NLR) was 6.2 (range 1–35) and 24-h LMR (24h_LMR) was 1.7 (range 0.3–5).

### Dynamic change and association between NLR and LMR

**Fig. 1:**
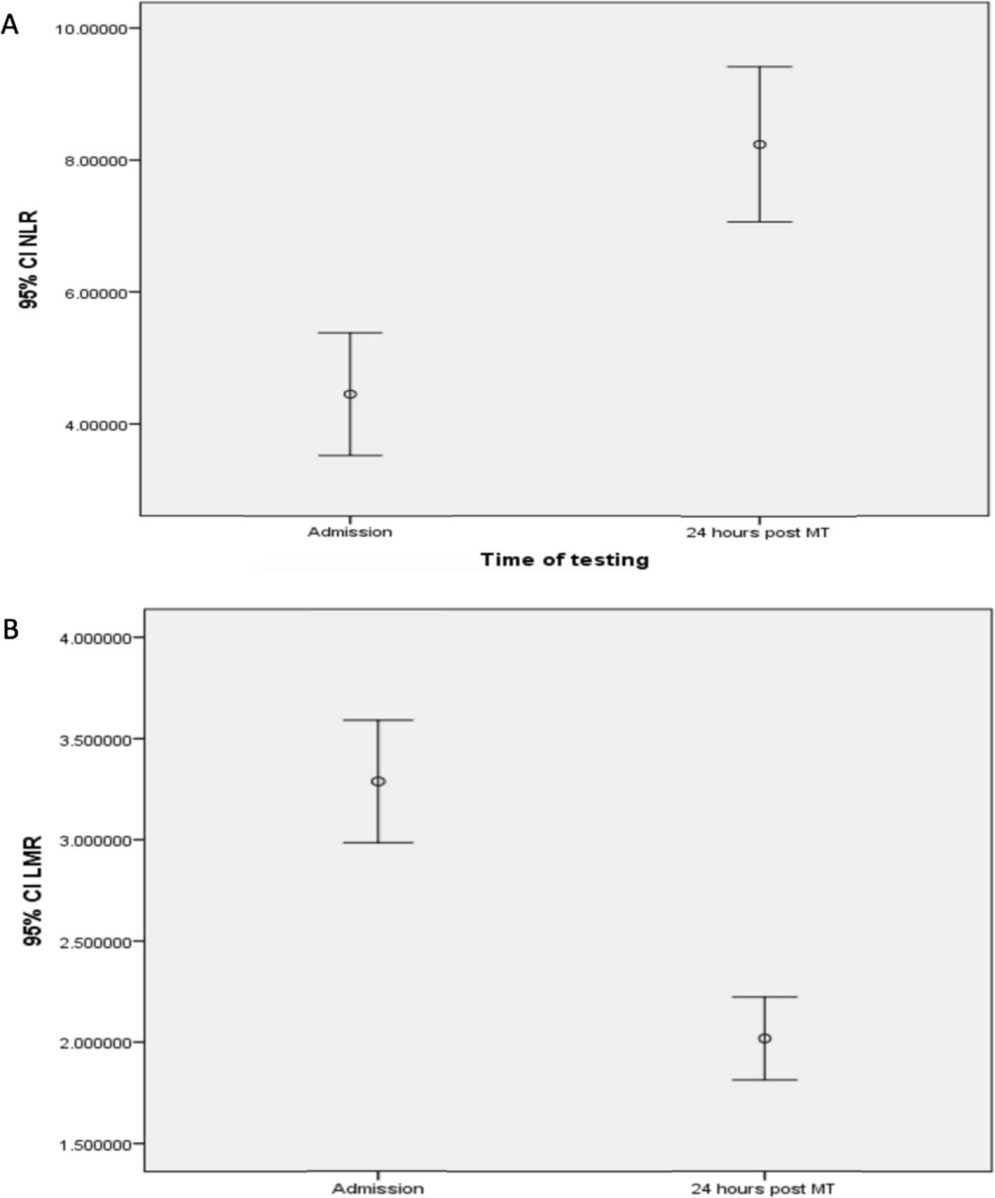
Dynamics of NLR (**a**) and LMR (**b**) from admission to 24 h after mechanical thrombectomy. An axis *y* figures reflects 95% confidence interval (Cl) that is a range of values 95% certain contains the true mean of the NLR and LMR

An increasing trend in the NLR (Fig. 1a) and decreasing trend in the LMR (Fig. 1b) was observed after 24-h post-MT, and there was correlation between admission NLR and LMR (*r* = − 7.47, *p* < 0.0001) and NLR and LMR 24 h after MT (*r* = − 6.69, *p* < 0.0001), respectively (Additional file 2: Figure S1 A and B).

Seventy-five percent cases had the combined rising NLR and falling LMR trend (the remaining 25% included those with unchanging NLR, rising NLR and LMR, or missing values). There was no significant association of a dynamic change in NLR or LMR and whether recanalisation was achieved. Eighty-three percent (101/121) of stroke patients with complete recanalisation had a rising NLR compared with 74% (23/31) of stroke patients with incomplete or no recanalisation (*χ*2 = 1.41; *p* = 0.23). Eighty-one percent (105/121) of stroke patients with complete recanalisation had a falling LMR compared with 81% (25/31) in those with incomplete or no recanalisation (*χ*2 = 0.7; *p* = 0.38).

### Correlation between NLR or LMR and the ischaemic area as identified using the NIHSS

**Fig. 2:**
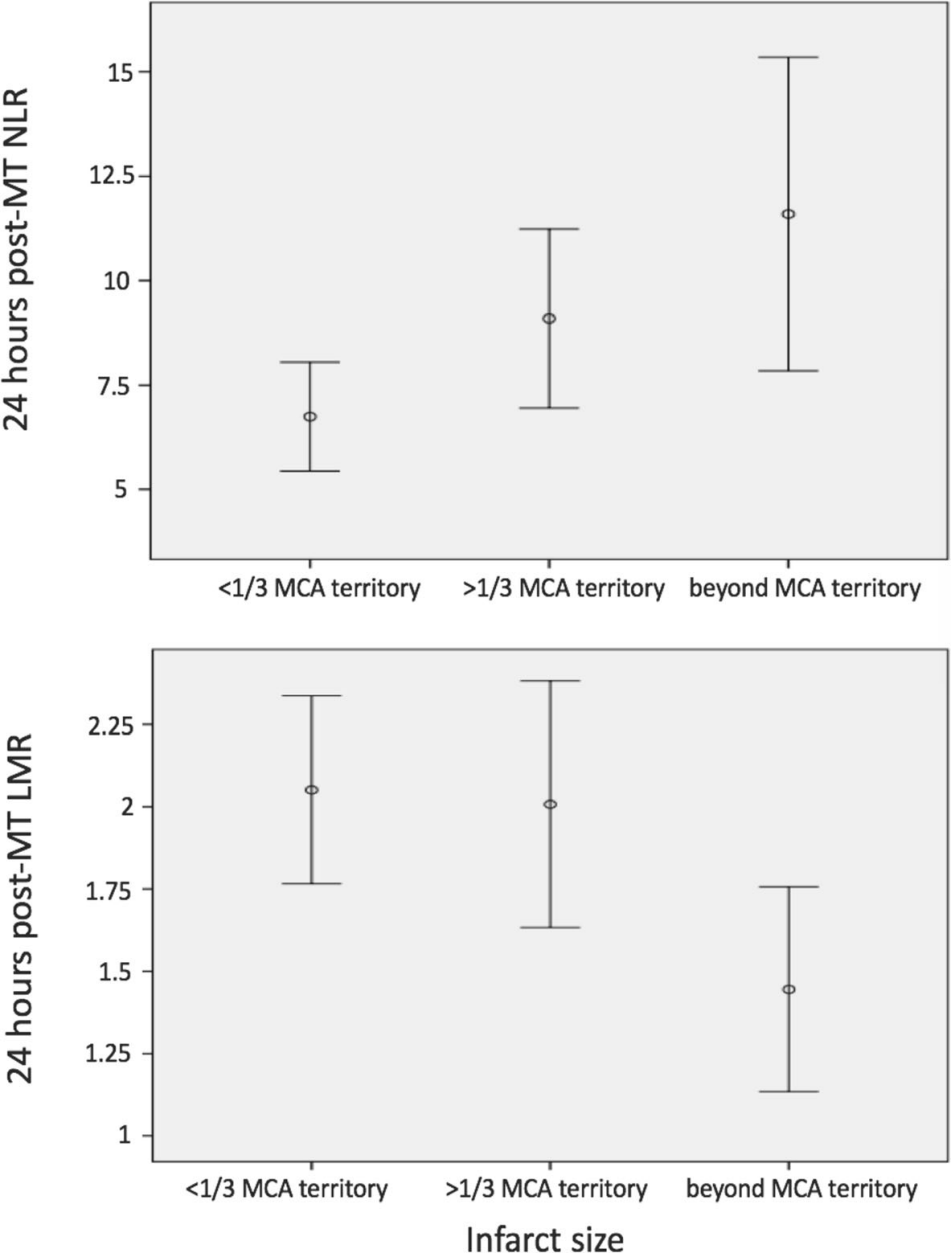
Correlation between LMR, NLR and infarct size based on The Alteplase Thrombolysis for Acute Noninterventional Therapy in Ischemic Stroke (ATLANTIS)/CT Summit criteria [14–16]

There was neither significant correlation between a_NLR or a_LMR and infarct size, nor between NLR or LMR and sICH, baseline NIHSS score or recanalisation on univariate analysis. However, higher 24h_NLR and lower 24h_LMR were associated with larger infarct size, *r* = 0.25, *p* = 0.008 and *r* = − 0.18, *p* = 0.05, respectively on univariate analysis (Fig. 2).

### NLR and LMR measured after the procedure were more correlated with long-term outcome

**Fig. 3:**
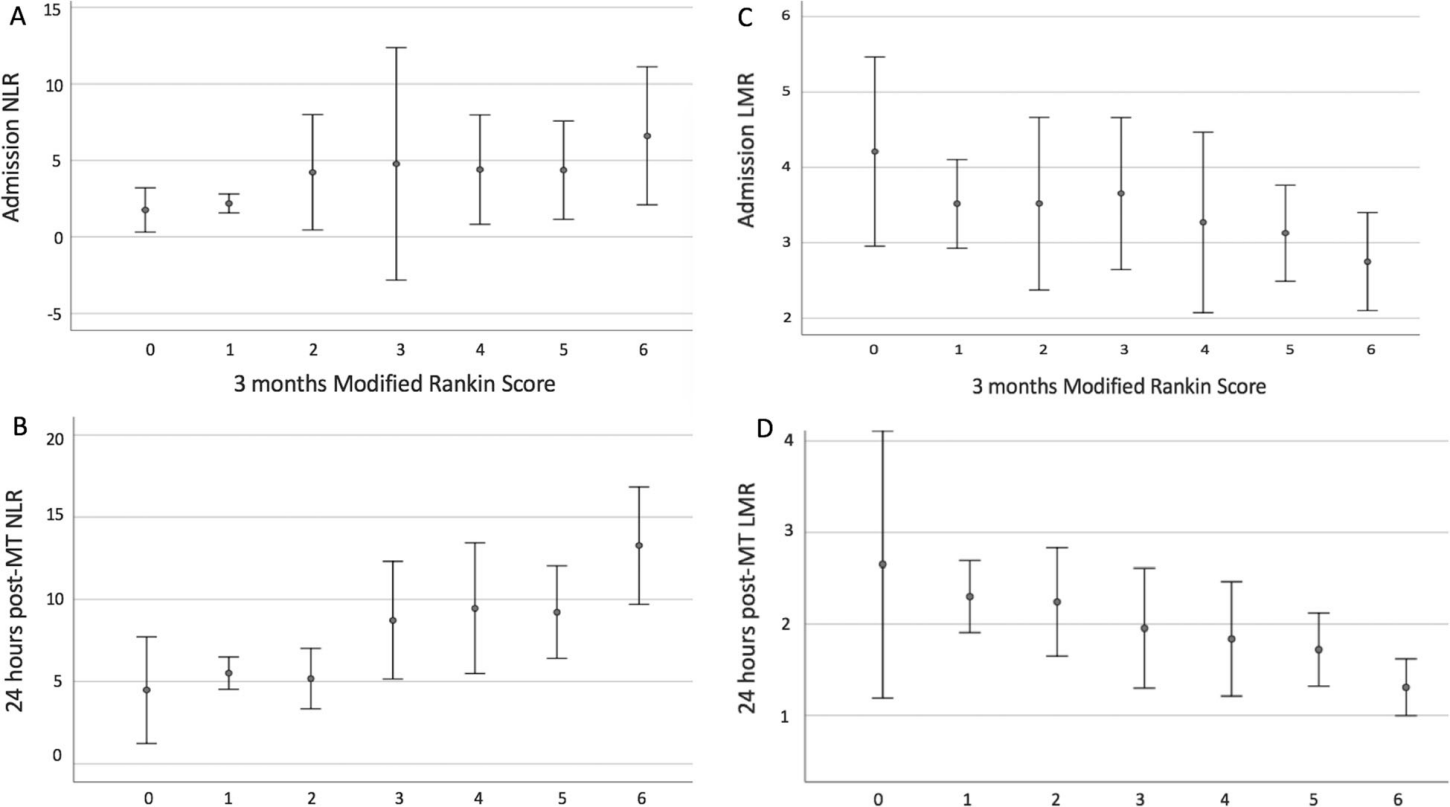
Correlation between NLR (**a** and **b**), LMR (**c** and **d**) and outcome

Higher a_NLR and 24h_NLR were associated with outcome as measured by 3-month mRS with there being poorer outcome on univariate analysis, *r* = 0.27, *p* = 0.055 and *r* = 0.47, *p* < 0.0001, respectively (Fig. 3a, b).

In a similar fashion, the a_LMR and 24h_LMR were associated with poorer outcome on univariate analysis but unlike the NLR, it was lower LMR that correlated, *r* = − 0.2, *p* = 0.01 and *r* = − 0.4, *p* < 0.0001, respectively (Fig. 3c, d).

### NLR and LMR association with an infarct size on multivariate logistic regression

The association noted above between 24h_NLR or 24h_LMR and infarct size weakened after age, baseline NIHSS, presence of good collateral supply, recanalisation and sICH adjustment on multivariate logistic regression (OR 0.011, 95% Cl − 0.002‑0.024, *p* = 0.099 and OR 0.018, 95% Cl − 0.067‑0.103, *p* = 0.674, respectively) (Additional file 1: Table S1).

### 24h_NLR and 24h_LMR association with poor outcome on multivariate logistic regression

Higher 24h_NLR as a continuous variable remained a significant predictor of poor outcome with an adjusted odds ratio (OR) of 0.022 (95% CI 0.009–0.34, *p* = 0.001) whereas the association between a_NLR and outcome noted above weakened (*p* = 0.059) when controlling for age, baseline NIHSS, infarct size, presence of good collateral supply, recanalisation and sICH on multivariate logistic regression (Additional file 1: Table S2). In this model, incomplete or absent recanalisation (mTICI 0–2a) was also significantly associated with poor outcome (OR 0.207, 95% CI 0.014–0.399, *p* = 0.036).

Similarly, lower values of 24h_LMR were strongly associated with poor outcome (adjusted OR − 0.093, 95% CI (− 0.175)−(− 0.012), *p* = 0.025) in contrast to the weak association between a_LMR (noted above) and outcome (*p* = 0.3) when controlling for age, baseline NIHSS, infarct size, presence of good collateral supply, recanalisation and sICH on multivariate logistic regression (Additional file 1: Table S3).

### 24h_NLR and 24h_LMR cut-off points distinguishing poor outcome

**Fig. 4:**
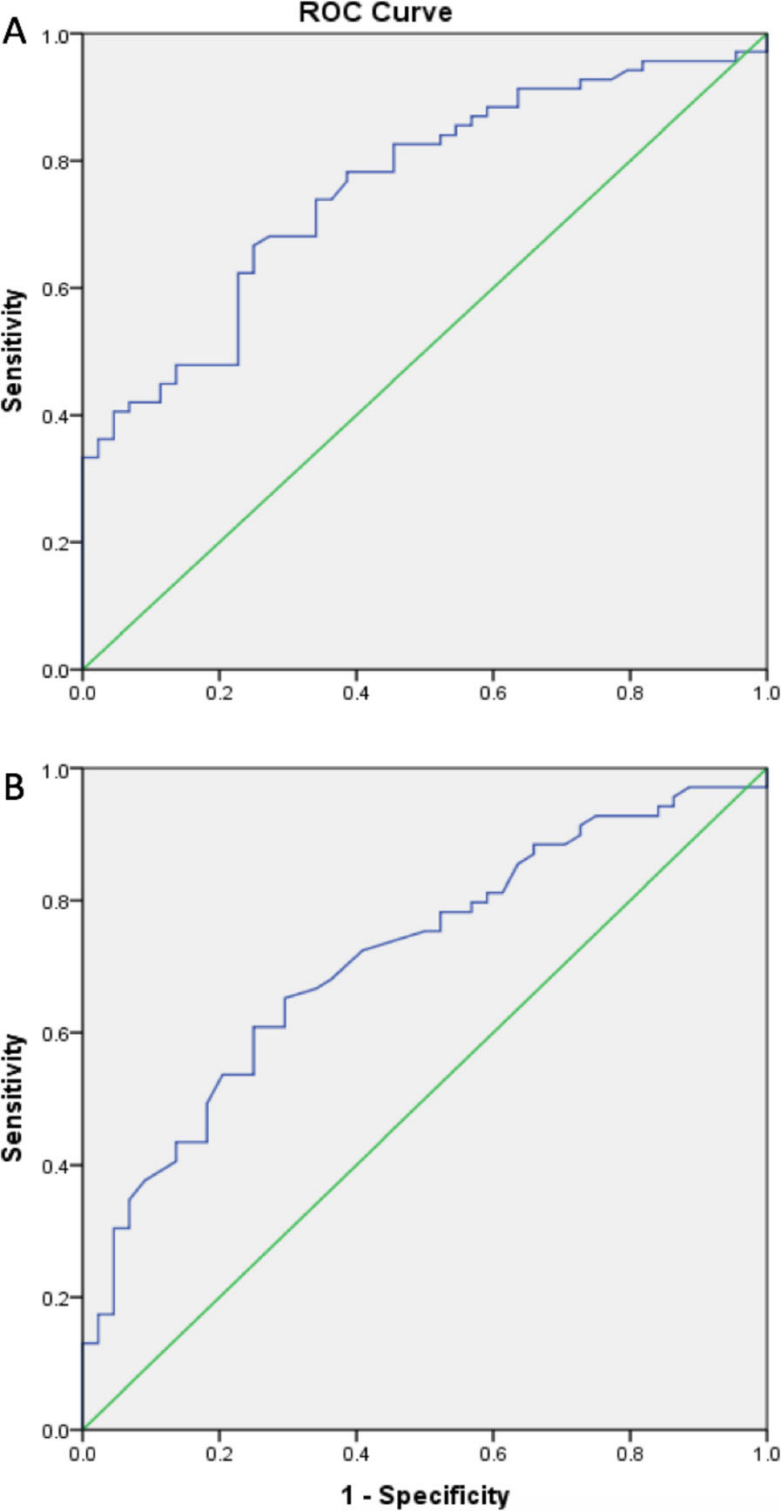
NLR (**a**) and LMR (**b**) receiver operating characteristic curve analysis

Receiver operating characteristic (ROC) curves were used to test the overall discriminative ability of the 24h_NLR and 24h_LMR for outcome and to establish optimal cut-off points at which the sum of the specificity and sensitivity was highest. The optimal cut-off values of the NLR and LMR that best discriminated poor outcome were 5.5 (80% sensitivity and 60% specificity) and 2.0 (80% sensitivity and 50% specificity) 24 h after MT respectively (Fig. 4).

The patients with high 24h_NLR were older (59.8 ± 10.5 vs 69.6 ± 12.5 years, *p* = 0.001) and had a higher proportion of atrial fibrillation (22 vs 44%, *p* = 0.01) (Table 1).

**Table 1:** Comparisons of baseline characteristics and outcomes between 24h_NLR groups

Characteristics^a^	NLR < 5.5 (*n* = 41)	NLR ≥ 5.5 (*n* = 72)	*p* value
Age, years (SD)	59.8 (10.5)	69.6 (12.5)	*p* = 0.001
Female, No. (%)	19 (46)	39 (54)	*p* = 0.39
Hypertension, No. (%)	17 (41)	41 (57)	*p* = 0.11
High cholesterol, No. (%)	10 (24)	23 (32)	*p* = 0.39
Ischaemic heart disease, No. (%)	2 (5)	9 (13)	*p* = 0.18
Congestive heart failure, No. (%)	1 (2)	9 (13)	*p* = 0.07
Atrial fibrillation^b^, No. (%)	9 (22)	32 (44)	*p* = 0.01
History of stroke/TIA, No. (%)	10 (24)	13 (18)	*p* = 0.42
Smoking, No. (%)	13 (32)	18 (25)	*p* = 0.44
Baseline NIHSS score, mean (range)	18 (4–27)	19 (2–28)	*p* = 0.121
Baseline mRS ≥ 1, No. (%)	9 (22)	17 (24)	*p* = 0.84
Intravenous thrombolysis, No (%)	34 (83)	55 (76)	*p* = 0.41
mTICI 2b/3, No (%)	35 (85)	50 (69)	*p* = 0.059
sICH, No (%)	1 (2)	8 (11)	*p* = 0.101
mRS at 3 months, mean (range)	2 (0–6)	4 (0–6)	*p* < 0.0001

*mRS* modified Rankin Scale, *NIHSS* National Institutes of Health Stroke Scale, *24h_NLR* neutrophil-lymphocyte ratio at 24 h after mechanical thrombectomy, *No* number, *TIA* transient ischaemic attack, *mTICI* modified thrombolysis in cerebral infarction, *sICH* symptomatic intracranial haemorrhage

^a^8 cases excluded with missing 24h_NLR data not included, 113 cases included

^b^Atrial fibrillation diagnosed from an admission 12 lead ECG

In contrast, the patients with low 24h_LMR were younger (72 ± 1.5 vs 57 ± 12.5, *p* < 0.0001), had a higher proportion of hypertension (36 vs 62%, *p* = 0.006), a higher baseline NIHSS score (17 vs 19, *p* = 0.026) and a poorer baseline mRS (6 vs 20%, *p* = 0.046) (Table 2).

**Table 2:** Comparisons of baseline characteristics and outcomes between 24h_LMR groups

Characteristics^a^	LMR < 2.0 (*n* = 68)	LMR ≥ 2.0 (*n* = 45)	*p* value
Age, years (SD)	57 (12.5)	72 (1.5)	*p* < 0.0001
Female, No. (%)	34 (50)	24 (53)	*p* = 0.72
Hypertension, No. (%)	42 (62)	16 (36)	*p* = 0.006
High cholesterol, No. (%)	22 (32)	11 (24)	*p* = 0.36
Ischaemic heart disease, No. (%)	8 (12)	3 (7)	*p* = 0.37
Congestive heart failure, No. (%)	8 (12)	2 (4)	*p* = 0.17
Atrial fibrillation^b^, No. (%)	29 (43)	12 (27)	*p* = 0.08
History of stroke/TIA, No. (%)	14 (21)	9 (20)	*p* = 0.93
Smoking, No. (%)	16 (24)	15 (33)	*p* = 0.25
Baseline NIHSS score, mean (range)	19 (2–28)	17 (4–25)	*p* = 0.026
Baseline mRS ≥ 1, No. (%)	20 (29)	6 (13)	*p* = 0.046
Intravenous thrombolysis, No (%)	54 (79)	35 (78)	*p* = 0.83
mTICI 2b/3, No (%)	47 (69)	38 (84)	*p* = 0.06
sICH, No (%)	8 (12)	1 (2)	*p* = 0.06
mRS at 3 months, mean (range)	4 (0–6)	2.5 (0–6)	*p* = 0.0003

*mRS* modified Rankin Scale, *NIHSS* National Institutes of Health Stroke Scale, *24h_LMR* lymphocyte-monocyte ratio at 24 h after mechanical thrombectomy, *No* number, *TIA* transient ischaemic attack, *mTICI* modified thrombolysis in cerebral infarction, *sICH* symptomatic intracranial haemorrhage

^a^8 cases excluded with missing 24h_LMR data not included, 113 cases included

^b^Atrial fibrillation diagnosed from an admission 12 lead ECG

## Discussion

Our study shows that a higher NLR and lower LMR tested 24 h after MT were independent predictors of 3-month poor functional outcome after MT for acute anterior circulation large vessel occlusion stroke.

NLR is a composite marker of absolute peripheral neutrophil and lymphocyte counts, and LMR is a composite marker of absolute peripheral lymphocyte and monocytes counts. These cells comprise the total leukocyte count which has previously been shown to be associated with cardiovascular and cancer mortality, as well as all-cause mortality [17–22]. However, they play a different role in the inflammation and possibly in the pathogenesis of these differing medical conditions. For instance, high neutrophil counts have been associated with adverse prognosis, whereas high lymphocyte counts have been considered to have protective effects on survival in cardiovascular patients [23–25]. While analysing them together may not highlight the opposing roles they seem to have, analysing them apart may miss the interaction between these subtypes and their association with different medical conditions. Indeed, among patients with acute myocardial infarction, it has been shown that an increased NLR is a predictor of in-hospital mortality and morbidity [26], and impaired myocardial perfusion after percutaneous coronary angioplasty [27]. Similarly, LMR has been reported to be associated with adverse prognosis in multiple malignancies [22, 28] and coronary artery disease [21, 29].

High admission NLR has been found to predict functional independence or death independent of age, treatment with IV rtPA and recanalisation [4]. Interestingly, baseline or admission NLR or LMR had no independent predictive value for outcome in our cohort presumably because the thrombectomy treatment modified the outcome. Intraparenchymal perivascular neutrophil migration occurs within 6 to 24 h [30, 31], and further accumulation of neutrophils in ischaemic and reperfused areas occurs at a higher rate after endovascular recanalisation and correlates with poor neurological outcome and brain damage severity both in humans and rodents [32]. Therefore, dynamic measurement of NLR or LMR may be a stronger predictive tool for outcome compared with single measurements. Higher NLR within 3 days after the stroke onset was previously associated with unfavourable functional outcome at discharge [33]. To our knowledge, dynamic NLR was not previously assessed in stroke patients treated with MT.

Previous studies suggested that the initial NLR was associated with mortality and infarct size in ischaemic stroke patients [34, 35]. However, there was no independent association between 24h_NLR or 24h_LMR and infarct size in our cohort. This may be related to evaluation of the post procedure CT scan performed in our study. Diffusion-weighted imaging measures performed after endovascular treatment were not included into our analysis. Previous studies reported a correlation between stroke severity and NLR determined at admission [36, 37]. We could not confirm these findings. Compared with previous studies [38, 39], we did not find association between NLR and sICH in spite of higher rate of sICH in our cohort.

Lower LMR after AIS has been associated with worse outcomes [40, 41]. The cut-off value of LMR that predicted poorer outcome in our cohort was lower compared with the previous studies (< 2.0 vs > 2.99) [10, 41]. LMR was previously assessed in AIS patients treated with thrombolytic therapy [10] but not in relation with endovascular cerebral treatment. In our cohort, lower LMR tested 24 h after MT was an independent predictor of 3-month poor functional outcome after MT for acute anterior circulation large vessel occlusion stroke independent of sICH.

Post-stroke inflammation has a dual role in ischaemic stroke. Peripheral immune cells are activated after stroke and may in turn influence the fate of ischaemic brain tissue [42]. Neutrophils respond early after stroke and indicate an active inflammatory reaction, while lymphocytes may have a regulatory function in inflammation inducing neuroprotection [42]. There is evidence that neutrophilia may provoke poor functional outcome in patients with good collaterals achieving successful reperfusion after MT. [43] Therefore, reduction of neutrophils and induction of lymphocyte after MT may improve functional outcome of AIS after MT.

Our data should be interpreted with some caution due to limitations of the study. These include retrospective bias inherent to the study design and a small sample size.

## Conclusion

This study suggests that NLR and LMR tested at 24 h after endovascular recanalisation therapy may reliably predict 3-month functional outcome. Based on our findings and previous studies, NLR and LMR may have utility as an inclusion criterion for future endovascular therapy clinical trials, and also suggest further exploration on modulating immune response to treat AIS.

## Supplementary information

**Additional file 1: Table S1.** Logistic regression 24h_NLR and 24h_LMR model of infarct size. **Table S2.** Logistic regression 24h_NLR and a_NLR model of mRS 3–6 at 3 months. **Table S3.** Logistic regression 24h_LMR and a_LMR model of mRS 3–6 at 3 months.

**Additional file 2: Figure S1.** Correlation between admission and 24-h after MT NLR (A) and LMR (B).

## Data Availability

Available

## Abbreviations

24h_LMR: 24 h post-mechanical thrombectomy lymphocyte-monocyte ratio
24h_NLR: 24 h post-mechanical thrombectomy neutrophil-lymphocyte ratio
a_LMR: Admission lymphocyte-monocyte ratio
a_NLR: Admission neutrophil-lymphocyte ratio
AIS: Acute ischaemic stroke
ATLANTIS: The Alteplase Thrombolysis for Acute Noninterventional Therapy in Ischemic Stroke
CT: Computed tomography
ECASS: European Cooperative Acute Stroke Study
ICH: Intracranial haemorrhage
IV: Intravenous
LMR: Lymphocyte-monocyte ratio
MCA: Middle cerebral artery
mRS: Modified Rankin score
MT: Mechanical thrombectomy
mTICI: Modified Thrombolysis in Cerebral Infarction
NIHSS: National Institutes of Health Stroke Scale
NLR: Neutrophil-lymphocyte ratio
ROC: Receiver operating characteristic
rtPA: Recombinant tissue plasminogen activator
sICH: Symptomatic intracranial haemorrhage
