# Supplementary material for: The association of neutrophil-lymphocyte ratio and lymphocyte-monocyte ratio with 3-month clinical outcome after mechanical thrombectomy following stroke
Source: J Neuroinflammation. 2020 Feb 18;17:60. doi: 10.1186/s12974-020-01739-y (PMC7026966; doi:10.1186/s12974-020-01739-y)
Supplement: Supplementary file 1 — Additional file 1: Table S1. Logistic regression 24h_NLR and 24h_LMR model of infarct size. Table S2. Logistic regression 24h_NLR and a_NLR model of mRS 3–6 at 3 months. Table S3. Logistic regression 24h_LMR and a_LMR model of mRS 3–6 at 3 months. [file 12974_2020_1739_MOESM1_ESM.docx]

**THE ASSOCIATION OF NEUTROPHIL-LYMPHOCYTE RATIO AND LYMPHOCYTE-MONOCYTE RATIO WITH 3-MONTH CLINICAL OUTCOME AFTER MECHANICAL THROMBECTOMY FOLLOWING STROKE**

Danielle Lux^1†^, Vafa Alakbarzade^2†^, Luke Bridge^1^, Camilla N. Clark^1^, Brian Clarke^1^, Liqun Zhang^1^, Usman Khan^1^, Anthony C. Pereira^1^

**SUPPLEMENTARY DATA**

| **Supplementary Table 1:** Logistic regression 24h_NLR and 24h_LMR model of infarct size* | | | | |
| --- | --- | --- | --- | --- |
|  | 24h_NLR association with infarct size | | 24h_LMR association with infarct size | |
|  | OR (95% Cl) | *p* value | OR (95% Cl) | *p* value |
| Age** | -0.030 (-0.239-0.178) | 0.774 | 0.022 (-0.186-0.230) | 0.833 |
| Baseline NIHSS score | 0.011 (-0.004-0.026) | 0.164 | 0.012 (-0.004-0.027) | 0.129 |
| Collateral supply*** | 0.206 (0.033-0.379) | 0.020 | 0.240 (0.064-0.416) | 0.008 |
| mTICI (0–2a vs 2b/3) | 0.379 (0.189-0.569) | 0.000 | 0.383 (0.189-0.577) | 0.000 |
| sICH | 0.407 (0.096-0.717) | 0.011 | 0.463 (0.154-0. 772) | 0.004 |
| 24h_NLR | 0.011 (-0.002—0.024) | 0.099 | 0.018 (-0.067—0.103) | 0.674 |
| *<1/3 MCA territory vs >1/3 MCA territory  **>80 vs <80  ***Good vs poor^12^  mRS, modified Rankin Scale; MCA, middle cerebral artery; NIHSS, National Institute of Health Stroke Scale; 24h_NLR, neutrophil lymphocyte ratio at 24 hours after mechanical thrombectomy; 24h_LMR, lymphocyte monocyte ratio at 24 hours after mechanical thrombectomy; mTICI, modified thrombolysis in cerebral infarction; sICH, symptomatic intracranial haemorrhage | | | | |

| **Supplementary Table 2:** Logistic regression 24h_NLR and a_NLR model of mRS 3–6 at 3 months | | | | |
| --- | --- | --- | --- | --- |
|  | 24h_NLR association with mRS 3–6 at 3 months | | a_NLR association with mRS 3–6 at 3 months | |
|  | OR (95% Cl) | *p* value | OR (95% Cl) | *p* value |
| Age* | 0.048 (-0.149-0.245) | 0.632 | 0.124 (-0.068-0.315) | 0.204 |
| Baseline NIHSS | 0.029 (0.014-0.043) | 0.000 | 0.025 (0.012-0.039) | 0.000 |
| Infarct size** | 0.060 (-0.123-0.242) | 0.518 | 0.097 (-0.035-0.228) | 0.149 |
| Collateral supply*** | 0.040 (-0.127-0.207) | 0.637 | 0.078 (-0.099-0.255) | 0.384 |
| mTICI (0–2a vs 2b/3) | 0.207 (0.014-0.399) | 0.036 | 0.182 (-0.027-0.391) | 0.087 |
| sICH | 0.226 (-0.077-0.529) | 0.142 | 0.259 (-0.026-0.544) | 0.074 |
| 24h_NLR | 0.022 (0.009—0.034) | 0.001 | 0.016 (-0.001—0.034) | 0.059 |
| *>80 vs <80  **<1/3 MCA territory vs >1/3 MCA territory  ***Good vs poor^12^  mRS, modified Rankin Scale; MCA, middle cerebral artery; NIHSS, National Institute of Health Stroke Scale; 24h_NLR, neutrophil lymphocyte ratio at 24 hours after mechanical thrombectomy; a_NLR, admission neutrophil lymphocyte ratio; mTICI, modified thrombolysis in cerebral infarction; sICH, symptomatic intracranial haemorrhage | | | | |

| **Supplementary Table 3:** Logistic regression 24h_LMR and a_LMR model of mRS 3–6 at 3 months | | | | |
| --- | --- | --- | --- | --- |
|  | 24h_LMR association with mRS 3–6 at 3 months | | a_LMR association with mRS 3–6 at 3 months | |
|  | OR (95% Cl) | *p* value | OR (95% Cl) | *p* value |
| Age* | 0.090 (-0.109-0.290) | 0.371 | 0.125 (-0.071-0.321) | 0.211 |
| Baseline NIHSS | 0.028 (0.013-0.043) | 0.000 | 0.027 (0.013-0.040) | 0.000 |
| Infarct size** | 0.118 (-0.067-0.303) | 0.210 | 0.112 (-0.076-0.300) | 0.239 |
| Collateral supply*** | 0.037 (-0.138-0.211) | 0.679 | 0.125 (-0.048-0.297) | 0.155 |
| mTICI (0–2a vs 2b/3) | 0.154 (0.046-0.353) | 0.130 | 0.197 (0.000-0.394) | 0.050 |
| sICH | 0.271 (-0.038-0.580) | 0.085 | 0.248 (-0.041-0.536) | 0.091 |
| 24h_NLR | -0.093 (-0.175—0.012) | 0.025 | -0.024 (-0.071—0.023) | 0.307 |
| *>80 vs <80  **<1/3 MCA territory vs >1/3 MCA territory  ***Good vs poor^12^  mRS, modified Rankin Scale; MCA, middle cerebral artery; NIHSS, National Institute of Health Stroke Scale; 24h_LMR, lymphocyte monocyte ratio at 24 hours after mechanical thrombectomy; a_LMR, admission lymphocyte monocyte ration; mTICI, modified thrombolysis in cerebral infarction; sICH, symptomatic intracranial haemorrhage | | | | |
