# Supplementary figures and images for: The association of neutrophil-lymphocyte ratio and lymphocyte-monocyte ratio with 3-month clinical outcome after mechanical thrombectomy following stroke
Source: J Neuroinflammation. 2020 Feb 18;17:60. doi: 10.1186/s12974-020-01739-y (PMC7026966; doi:10.1186/s12974-020-01739-y)

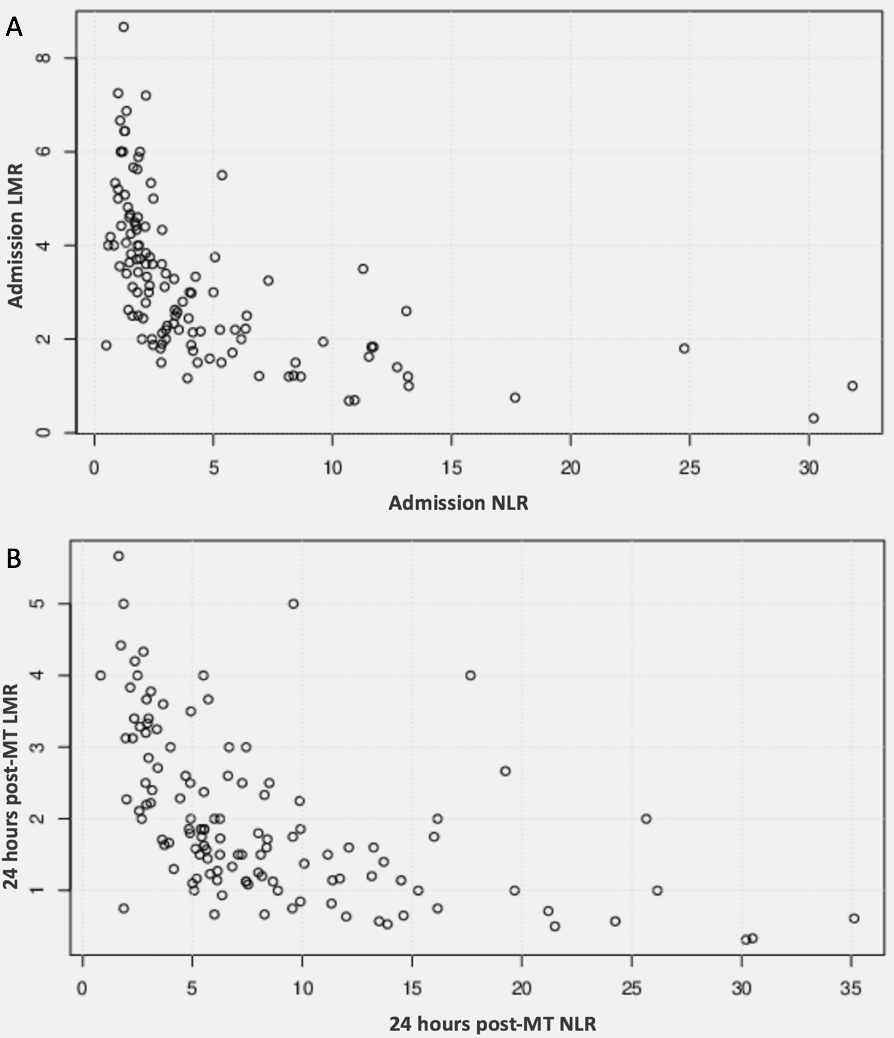

Supplement: Supplementary file 2 — Additional file 2: Figure S1. Correlation between admission and 24-h after MT NLR (A) and LMR (B). [file 12974_2020_1739_MOESM2_ESM.tiff]
